# A Pan‐Subtype Fatty Acid Metabolic Signature Reveals Universal Prognostic Stratification in Breast Cancer

**DOI:** 10.1002/cnr2.70427

**Published:** 2026-01-09

**Authors:** Yunjian Song, Jiayue Li, Meiyue Zhu, Yi Xia, Deqiang Wang, Zhenhua Sun, Liang Ou, Lei Yang

**Affiliations:** ^1^ Medical School of Nantong University Nantong China; ^2^ School of Public Health Nantong University Nantong China; ^3^ Comprehensive Cancer Center, Affiliated Hospital of Jiangsu University Zhenjiang China; ^4^ Department of Thyroid and Breast Surgery Affiliated Hospital of Jiangsu University Zhenjiang China; ^5^ Department of Oncology Tumor Hospital Affiliated to Nantong University Nantong China

## Abstract

**Background:**

Breast cancer exhibits profound molecular heterogeneity. Each subtype is characterized by distinct biological features, therapeutic responses, and prognostic outcomes. However, a unifying feature across most breast cancer subtypes is an adipocyte‐enriched microenvironment. Dysregulated lipid metabolism drives tumor progression across subtypes.

**Aims:**

We developed a universal prognostic model independent of molecular classification based on fatty acid metabolism‐related genes.

**Methods:**

Prognostic fatty acid metabolism‐related genes were identified in the training set using univariate Cox regression. LASSO regression refined these genes to construct the fatty acid metabolic signature for prognosis (FAMOUS), enabling risk stratification. FAMOUS's prognostic utility was validated in the testing set and external cohort, and across molecular subtypes and therapies.

**Results:**

FAMOUS comprises 15 genes. High FAMOUS scores independently predicted poor overall survival in the training set (HR = 4.97, 95% CI: 3.17–7.79; *p* < 0.001), testing set (HR = 7.39, 95% CI: 2.19–24.97; *p* = 0.001), and GSE72245 (HR = 7.97, 95% CI: 3.39–18.75; *p* < 0.001), after adjusting for molecular subtype, stage, and age. It stratified risk across Luminal A, Luminal B, HER2+, and TNBC subtypes and retained prognostic value in patients receiving chemotherapy, radiotherapy, endocrine therapy, or trastuzumab. High FAMOUS scores correlated with immune‐suppressive microenvironments, marked by downregulated immune‐related pathways and altered immune cell infiltration (e.g., reduced CD8+ T cells, enriched M2 macrophages), suggesting implications for immunotherapy patient stratification.

**Conclusion:**

FAMOUS is a novel, pan‐subtype prognostic tool for breast cancer, transcending molecular classification and therapeutic modalities.

## 1 Introduction

Breast cancer remains the leading cause of cancer‐related mortality in women worldwide, with its high incidence and mortality rates posing a critical public health burden. In 2022, approximately 2.3 million new cases and 666 000 deaths were reported globally [1], highlighting the profound societal impact of the disease. The disease spans all postpubertal age groups, with incidence rising progressively with age [2]. Breast cancer is clinically and molecularly heterogeneous. It is classified into distinct subtypes—Luminal A, Luminal B, HER2+, and triple‐negative breast cancer (TNBC)—each characterized by unique biological features and prognostic profiles that influence therapeutic efficacy [3].

Current management strategies, including surgery, chemotherapy, radiotherapy, endocrine therapy, targeted therapies, and immunotherapy, remain foundational to breast cancer care. However, their effectiveness varies substantially across subtypes. TNBC is characterized by aggressive behavior, limited therapeutic options, and poor outcomes, making it a prime example of the urgent need for precision‐driven innovations [4].

Precision medicine has underscored the importance of molecular biomarkers in guiding therapeutic decisions. Within this framework, metabolic reprogramming—particularly dysregulated fatty acid metabolism—has emerged as a hallmark of breast cancer pathogenesis [5]. The lipid‐rich mammary microenvironment facilitates dynamic crosstalk between tumor cells and adipocytes, driving carcinogenesis, proliferation, and therapy resistance [6]. Despite its therapeutic promise, our mechanistic understanding of how altered fatty acid metabolism influences tumor progression and treatment response remains limited, and targeted strategies targeting this pathway are still underdeveloped. Consequently, identifying robust fatty acid metabolism‐ associated biomarkers is critical for advancing subtype‐agnostic prognostic frameworks and therapeutic interventions.

Here, we developed a fatty acid metabolic signature for prognosis (FAMOUS) using transcriptomic data. FAMOUS‐based risk stratification demonstrated robust prognostic utility across all molecular subtypes and therapeutic modalities, offering a universal tool to refine clinical decision‐making.

## 2 Patients and Methods

### 2.1 Patients

Breast cancer patients were selected from The Cancer Genome Atlas (TCGA) and Gene Expression Omnibus (GEO) databases. Inclusion criteria included the availability of transcriptomic data for FAMOUS construction, documented molecular subtype classification, a pathologically confirmed diagnosis of breast cancer, and no prior exposure to radiotherapy, chemotherapy, targeted therapy, immunotherapy, or other anticancer treatments. Sequencing data and clinical annotations were obtained from TCGA (https://www.cbioportal.org/datasets) and GEO (https://www.ncbi.nlm.nih.gov/geo/) databases.

### 2.2 Gene Selection

Genes were curated from the KEGG_FATTY_ACID_METABOLISM, HALLMARK_FATTY_ACID_METABOLISM, and REACTOME_FATTY_ACID_ METABOLISM pathways. After deduplication, 156 genes were retained for analysis (Table S1).

### 2.3 FAMOUS Construction

We first applied the *ComBat* algorithm (from the *sva* R package) to the gene‐expression data to remove batch effects. The TCGA breast cancer cohort was stratified into training (70%) and validation (30%) sets via stratified randomization [7]. In the training set, prognostic genes linked to overall survival (OS) were identified using univariate Cox regression. Optimal expression thresholds for high/low gene expression subgroups were determined via the *Survminer* R package to maximize OS discrimination. Gene expression levels were binarized (0/1) relative to these thresholds [8]. A Cox regression model, regularized by the least absolute shrinkage and selection operator (LASSO), refined gene selection through penalized likelihood optimization. The FAMOUS score was calculated as: FAMOUS score = ∑ (Gene expression level × Cox regression coefficient). Prognostic performance was validated in the TCGA validation set and external cohort GSE72245 [9].

### 2.4 Gene Set Enrichment Analysis (GSEA)

Differentially expressed genes (DEGs) between low‐ and high‐FAMOUS‐score subgroups were identified (adjusted *p* < 0.05, |log2FC| > 1). Pathway enrichment was analyzed via GSEA using REACTOME, gene ontology (GO), and Kyoto Encyclopedia of Genes and Genomes (KEGG) databases, implemented in NetworkAnalyst 3.0 (https://www.networkanalyst.ca/).

### 2.5 Immune Infiltration Estimation

Immune cell abundance in the tumor microenvironment (TME) was estimated using the CIBERSORT algorithm with the LM22 gene signature (https://cibersortx.stanford.edu/), based on transcriptomic data.

### 2.6 Statistical Analysis

Group differences were assessed using χ^2^, Fisher's exact test, *t*‐tests, Mann–Whitney U tests, or Kruskal–Wallis tests, as appropriate. Survival outcomes were compared via Kaplan–Meier curves and log‐rank tests. Multivariate Cox regression evaluated the independent prognostic value of FAMOUS, with hazard ratios (HRs) and 95% confidence intervals (CIs) reported. A two‐sided *p* < 0.05 defined statistical significance. Analyses were performed in R (v4.3.2), Bioconductor packages, and SPSS (v19.0).

## 3 Results

### 3.1 Patient Characteristics

The TCGA breast cancer cohort included 1036 eligible patients, stratified into training (*n* = 725) and testing (*n* = 311) sets at a 7:3 ratio. Clinicopathological characteristics were balanced between sets (Tables S2 and S3). The cohort comprised 98.8% females (*n* = 1024) and 1.2% males (*n* = 12), with 30.5% elderly patients (≥ 65 years) and 24.5% stage III/IV cases. Molecular subtypes included Luminal A (46.5%, *n* = 482), Luminal B (18.2%, *n* = 189), HER2+ (6.9%, *n* = 72), and TNBC (15.4%, *n* = 160). Postoperative adjuvant chemotherapy, radiotherapy, and trastuzumab therapy were documented for 49.1% (*n* = 509), 47.5% (*n* = 492), and 6.9% (*n* = 71) of patients, respectively. The external GSE72245 validation cohort (*n* = 118) comprised Luminal A (21.2%, *n* = 25), Luminal B (27.1%, *n* = 32), HER2+ (25.4%, *n* = 30), and TNBC (26.3%, *n* = 31) cases (Table S4).

### 3.2 FAMOUS Construction and Validation

**FIGURE 1:**
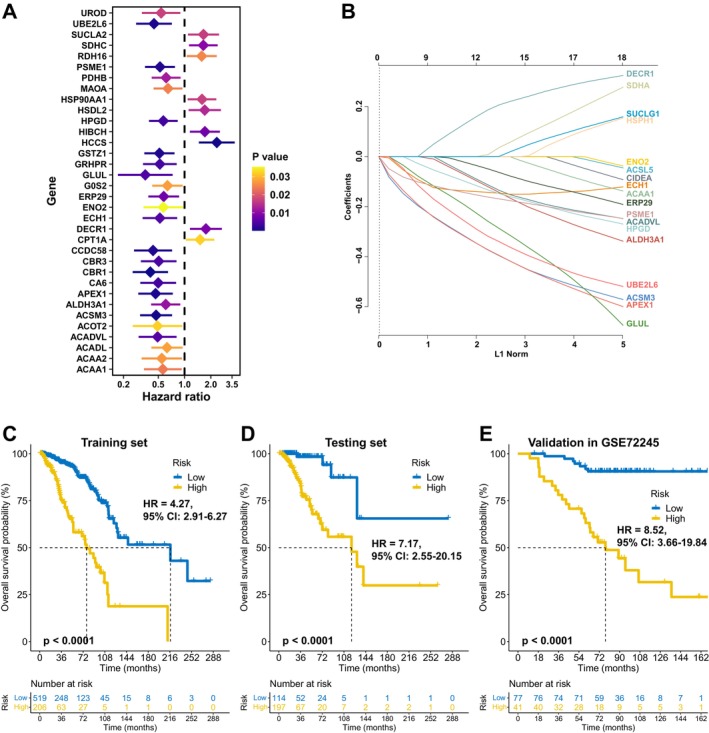
Development and validation of the FAMOUS signature. (A) Prognosis‐associated fatty acid metabolism genes identified through univariate Cox regression analysis (training set, *n* = 725). (B) LASSO coefficient profiles for gene selection in the Cox proportional hazards model (optimal λ determined by 10‐fold cross‐validation). (C–E): Kaplan–Meier survival curves demonstrating significant stratification of overall survival (OS) by FAMOUS risk groups in: (C) training set, (D) internal testing set, and (E) independent GSE72245 cohort. FAMOUS: A fatty acid metabolic signature for prognosis; LASSO: Least absolute shrinkage and selection operator.

In the TCGA training set, univariate Cox regression identified 34 prognostic fatty acid metabolism‐related genes (Figure 1A), of which 15 were selected via LASSO regression to construct FAMOUS (Figure 1B). The full names, functional links, regression coefficients in the model, and breast‐cancer–related references for these 15 genes are all listed in Table S5. Using the optimal OS‐related cutoff, FAMOUS stratified patients into high‐ and low‐risk groups. High‐risk patients exhibited significantly worse OS in the TCGA training set (Figure 1C) and testing set (Figure 1D). These findings were validated in the GSE72245 cohort (Figure 1E).

### 3.3 FAMOUS Score Is an Independent Prognostic Predictor

**FIGURE 2:**
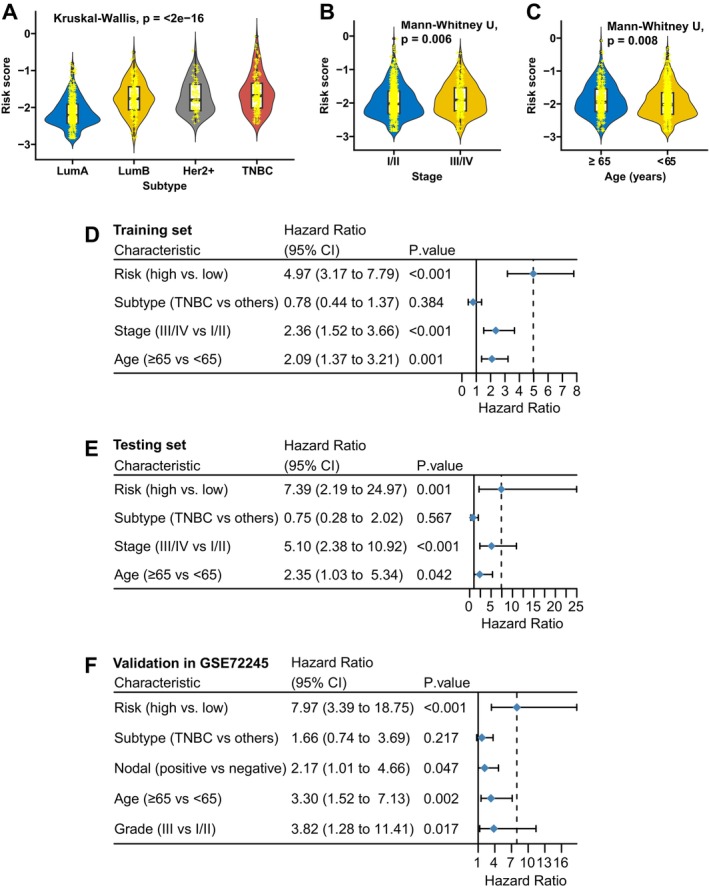
Multivariate prognostic validation of FAMOUS. (A–C) Association of FAMOUS scores with established clinicopathological parameters: (A) Molecular subtypes, (B) TNM staging, (C) patient age at diagnosis. (D–F) Multivariable Cox regression confirming FAMOUS as an independent OS predictor after adjusting for clinical covariates in: (D) Training set, (E) testing set, and (F) GSE72245 cohorts.

FAMOUS scores increased incrementally across subtypes (Luminal A < Luminal B < HER2+ < TNBC; Figure 2A) and correlated with advanced stage (stage III/IV vs. I/II: *p* = 0.006; Figure 2B) and older age (≥ 65 vs. < 65 years: *p* = 0.008; Figure 2C) in TCGA. In the GSE72245 cohort, FAMOUS score was also markedly higher in HER2‐positive and TNBC compared to the other two subtypes (Table S6). In multivariate analysis, FAMOUS remained an independent OS predictor in the TCGA training (HR = 4.97, 95% CI: 3.17–7.79; Figure 2D) and testing sets (HR = 7.39, 95% CI: 2.19–24.97; Figure 2E), validated in GSE72245 (HR = 7.97, 95% CI: 3.39–18.75; Figure 2F).

### 3.4 Pan‐Subtype and Pan‐Therapeutic Applicability of FAMOUS

**FIGURE 3:**
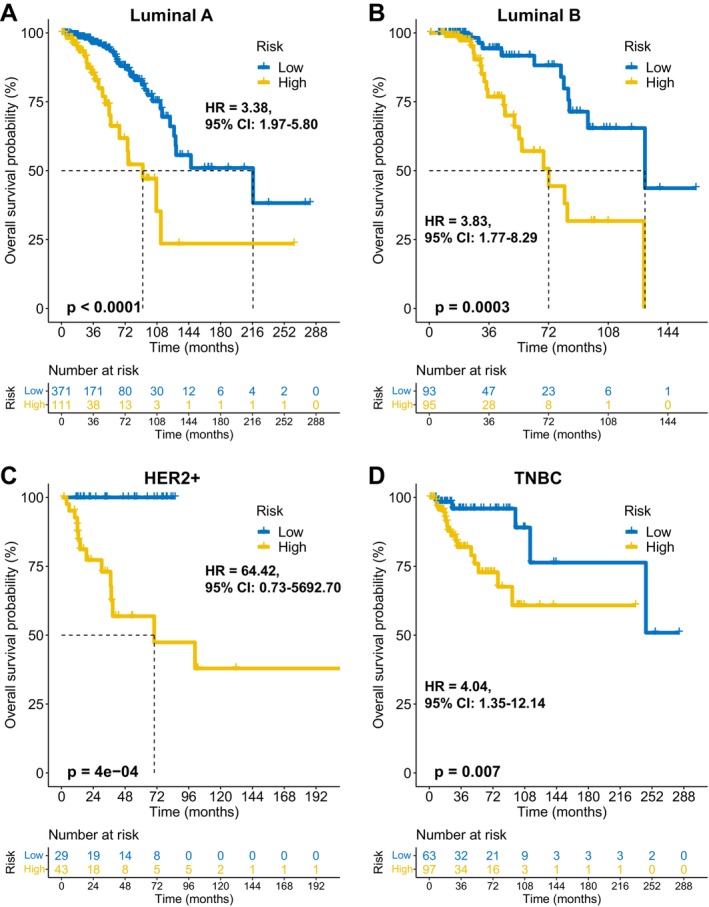
Subtype‐specific prognostic performance of FAMOUS in TCGA. (A–D) Kaplan–Meier analyses demonstrating FAMOUS‐mediated survival stratification within intrinsic subtypes: (A) Luminal A, (B) Luminal B, (C) HER2+, (D) TNBC. TCGA: The Cancer Genome Atlas; TNBC: Triple‐negative breast cancer.

**FIGURE 4:**
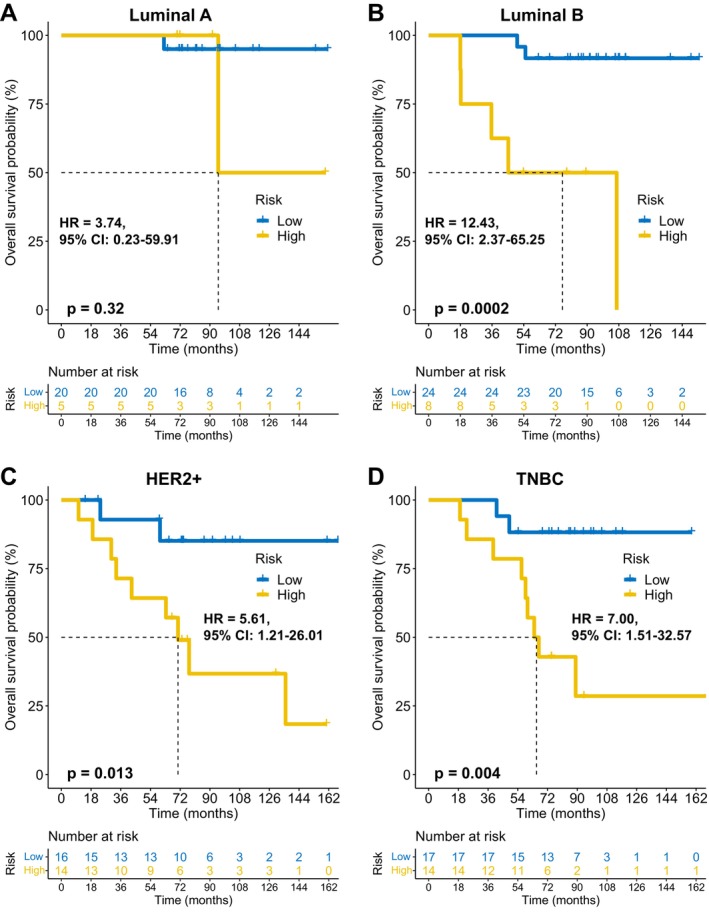
External validation of subtype prognostication of FAMOUS in GSE72245. (A–D) Consistent survival discrimination across molecular subtypes: (A) Luminal A, (B) Luminal B, (C) HER2+, and (D) TNBC.

**FIGURE 5:**
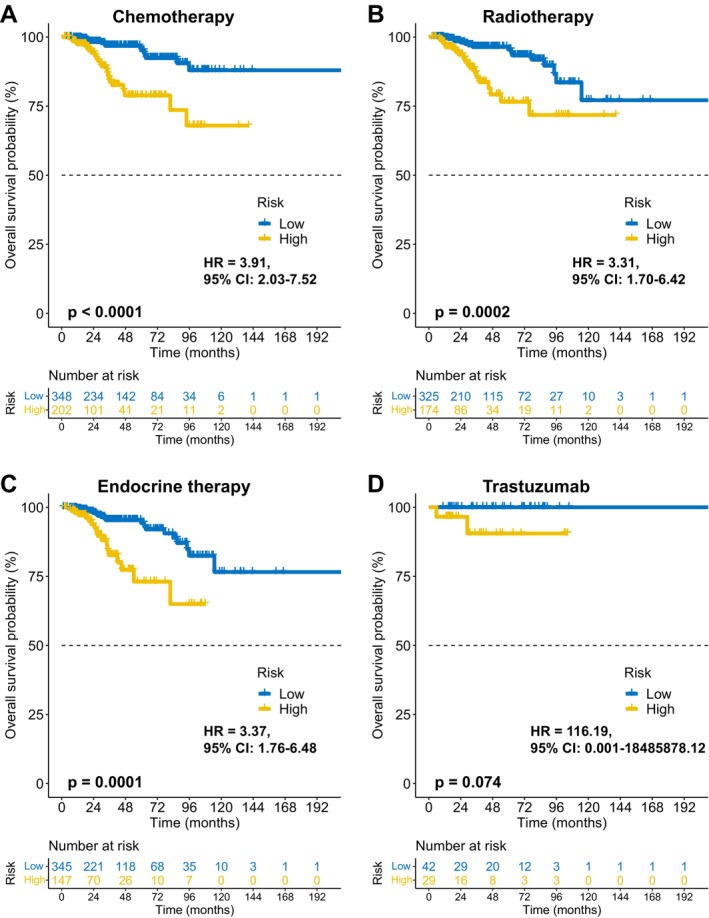
Therapeutic context‐independent prognostic value of FAMOUS. (A–D) FAMOUS maintains predictive capacity across treatment subgroups in TCGA: (A) chemotherapy, (B) radiotherapy, (C) endocrine therapy, and (D) Trastuzumab therapy.

FAMOUS significantly stratified OS risk across all molecular subtypes (Luminal A/B, HER2+, TNBC; *p* < 0.05; Figure 3A–D) in TCGA. These results were validated in the GSE72245 cohort (Figure 4A–D), though statistical significance was not reached in Luminal A due to limited sample size (Figure 4A). Besides, FAMOUS‐based risk stratification significantly predicted prognosis across therapies (chemotherapy, radiotherapy, endocrine therapy; *p* < 0.05; Figure 5A–C) in TCGA. In trastuzumab‐treated patients, a clinically relevant trend emerged despite limited statistical power (Figure 5D).

### 3.5 Immune Relevance of FAMOUS

**FIGURE 6:**
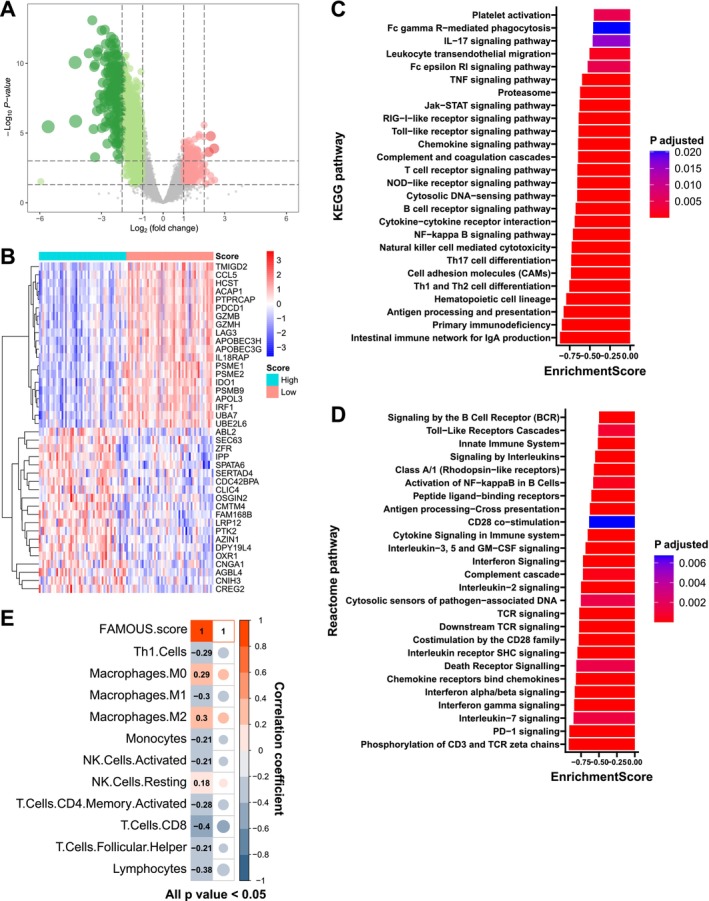
Immune relevance of FAMOUS in TNBC. (A) Volcano plot of 1079 differentially expressed genes (DEGs) between high and low FAMOUS score groups. (B) Heatmap for top 40 DEGs between high and low FAMOUS scores. (C) and (D): Selected pathways enriched by GSEA from (C) KEGG and (D) Reactome databases. (E) Correlations of FAMOUS score with immune cell abundance estimated by the CIBERSORT algorithm. KEGG: Kyoto Encyclopedia of Genes and Genomes.

In TNBC from TCGA, 1079 DEGs distinguished high‐ and low‐FAMOUS groups (Figure 6A and Table S7). Among the top 40 most significantly DEGs, immune‐related genes such as PDCD1 (PD‐1), LAG3, and GZMB were observed (Figure 6B). GSEA based on KEGG (Figure 6C) and Reactome (Figure 6D) revealed enrichment of immune activation pathways (e.g., antigen processing and presentation, T cell receptor signaling pathway, and interferon gamma signaling) in low‐FAMOUS groups, while high‐FAMOUS groups exhibited immune suppression (Table S8). Similar results were obtained from the biological process analysis of GO (Table S8). Consistent with these findings, FAMOUS score inversely correlated with immune‐activated cells such as CD8+ T cells and positively correlated with immune‐suppressive cells like M2 macrophages (Figure 6E). Similar immune trends were observed across all subtypes (Tables S9–S11), indicating FAMOUS reflects tumor immune microenvironment (“hotness”) polarization.

## 4 Discussion

Lipids are essential macromolecules that function as energy reservoirs, signaling mediators, and structural components of cellular membranes. Dysregulated lipid homeostasis promotes tumor progression by modulating therapeutic responses and metastatic potential [10]. Cancer cells exploit lipid metabolic pathways to modulate tumor‐stromal interactions and evade immune surveillance, thereby fostering a treatment‐resistant TME [11]. Here, we developed the FAMOUS score to quantify fatty acid metabolic activity in breast cancer. FAMOUS independently stratifies prognosis across molecular subtypes and therapies while revealing immune‐suppressive TME features, positioning it as a pan‐subtype prognostic tool with implications for immunotherapy.

The mammary stroma is uniquely enriched with lipid‐laden adipocytes that secrete fatty acids and adipocytokines in bioavailable forms. These peritumoral adipocytes promote metastatic competence by inducing lipidomic remodeling, activating epithelial‐mesenchymal transition (EMT), and enhancing pro‐survival signaling [12]. Although breast cancer subtypes exhibit distinct metabolic profiles, they largely rely on fatty acid metabolism for progression [13]. This convergence on lipid dysregulation—a shared mechanism of therapeutic resistance—underpins the ability of FAMOUS to transcend molecular heterogeneity and predict outcomes across different treatment paradigms.

Notably, FAMOUS captures the intricate interplay between fatty acid metabolism and immune evasion mechanisms. High FAMOUS scores are associated with reduced CD8+ T cell infiltration, increased M2 macrophage abundance, and suppression of immune activation pathways such as antigen presentation and interferon signaling. These findings align with the metabolic competition model, in which tumor cells outcompete immune cells for nutrients, thereby reshaping the TME into an immunosuppressive niche [14]. Thus, FAMOUS may serve as a tool to identify patients who are likely to benefit from immunotherapy, particularly in aggressive subtypes such as TNBC. However, the relationship between FAMOUS and immunotherapy efficacy still requires validation in clinical cohorts.

Immunotherapy has emerged as a cornerstone of breast cancer management, particularly following the approval of pembrolizumab combined with chemotherapy for TNBC. This regimen is now approved for both neoadjuvant and advanced settings, broadening therapeutic avenues for this aggressive subtype [15, 16, 17]. Recent trials, including KEYNOTE‐756 [18] and CheckMate 7FL [19], demonstrated that adding nivolumab or pembrolizumab to neoadjuvant chemotherapy significantly enhances pathological complete response rates in high‐risk, early‐stage ER+/HER2− patients, particularly those with elevated PD‐L1 expression. Nevertheless, immunotherapy efficacy remains suboptimal, with pronounced response heterogeneity across molecular subtypes [20, 21]. The FAMOUS score, which may reflect fatty acid metabolism‐driven immune modulation, exhibits pan‐subtype relevance and may refine patient selection for immunotherapy by identifying subtype‐specific populations poised to benefit.

The predictive power of the FAMOUS score is inseparable from the individual roles of the genes that compose its signature. ACAA1, for example, accelerates breast‐cancer cell proliferation by driving cell‐cycle progression via CDK4, thereby conferring resistance to the CDK4/6 inhibitor abemaciclib [22]. DECR1 is overexpressed in breast tumors and correlates with poor patient outcome; its knockdown markedly suppresses proliferation and migration by modulating ferroptosis [23]. Other genes in the signature, such as ALDH3A1, GLUL, and SDHA, have likewise been linked to breast‐cancer growth, drug resistance, or prognosis, although their precise mechanisms remain unclear [24, 25, 26]. For ACSM3, ECH1, and SUCLG1, no prior association with breast cancer has been reported. Collectively, these observations underscore that our understanding of fatty‐acid‐metabolism genes in breast cancer is still fragmentary; deeper investigation will not only illuminate tumor metabolism and progression but also reveal novel therapeutic targets.

This study has several limitations. First, FAMOUS was constructed using public datasets, and validation in proprietary cohorts is needed. In addition, the findings from retrospective analyses require prospective validation. Second, despite the large sample size used to construct FAMOUS, population heterogeneity may still affect its generalizability. Moreover, the molecular mechanisms by which the 15 genes comprising FAMOUS influence breast cancer are not fully understood. Some of these genes have not yet been reported in the context of breast cancer. Furthermore, it is necessary to validate the gene expression findings at the protein level and to incorporate mass spectrometry‐based metabolomics for quantitative profiling of fatty acid metabolites in tumor tissues. This would help bridge the gap between gene expression alterations and functionally relevant metabolic reprogramming. Finally, the clinical correlation between FAMOUS and immunotherapy outcomes remains to be investigated. The strengths of this study lie in the development of a universal breast cancer prognostic evaluation system.

In summary, our findings underscore the pan‐subtype influence of fatty acid metabolism on breast cancer progression and therapeutic responses, as quantified by the FAMOUS score. Future investigations should prioritize elucidating the mechanistic underpinnings of FAMOUS‐associated pathways, identifying druggable targets within this metabolic axis, developing multiomics‐integrated prognostic models, and advancing translational studies to bridge preclinical insights with clinical applications.

## Author Contributions

Y.S., J.L., D.W., Z.S., L.O., and L.Y. designed the study. D.W., Z.S., L.O., and L.Y. wrote the first draft of the manuscript. Y.S., J.L., M.Z., Y.X., D.W., Z.S., L.O., and L.Y. acquired data. Y.S., J.L., D.W., Z.S., L.O., and L.Y. analyzed the data. Y.S., J.L., D.W., Z.S., L.O., and L.Y. interpreted the data. L.Y. revised the manuscript.

## Funding

The study was funded by the Natural Science Foundation of Jiangsu Province (BK20231252) and the Key Medical Research Projects of Jiangsu Provincial Health Commission (K2023026).

## Ethics Statement

The study was conducted in accordance with the Declaration of Helsinki and approved by the Institutional Review Board of Affiliated Hospital of Jiangsu University (protocol code KY2021K0908 and September 2021).

## Conflicts of Interest

The authors declare no conflicts of interest.

## Supporting information


**Table S1:** Genes included for analysis.
**Table S2:** TCGA cohort.
**Table S3:** Clinicopathological features between the testing and training sets.
**Table S4:** GSE72245 cohort.
**Table S5:** The regression coefficients and details of the genes in FAMOUS.
**Table S6:** Clinicopathological features and FAMOUS score in GSE72245.
**Table S7:** DEGs between the high and low FAMOUS score groups in TNBC.
**Table S8:** GSEA results in TNBC.
**Table S9:** GSEA results in Luminal A subtype.
**Table S10:** GSEA results in Luminal B subtype.
**Table S11:** GSEA results in HER2+ subtype.

## Data Availability

All data relevant to the study that is not in the article and Supporting Information are available from the corresponding author on reasonable request.
